# Gamma Knife Radiosurgery for Indirect Dural Carotid–Cavernous Fistula: Long-Term Ophthalmological Outcome

**DOI:** 10.3390/life12081175

**Published:** 2022-08-01

**Authors:** Chiung-Chyi Shen, Yuang-Seng Tsuei, Meng-Yin Yang, Weir-Chiang You, Ming-His Sun, Meei-Ling Sheu, Liang-Yi Pan, Jason Sheehan, Hung-Chuan Pan

**Affiliations:** 1 Department of Neurosurgery, Taichung Veterans General Hospital, Taichung 40201, Taiwan; ccshen@vghtc.gov.tw (C.-C.S.); astrocytoma2001@yahoo.com.tw (Y.-S.T.); yangmy04@gamil.com (M.-Y.Y.); mhsun@vghtc.gov.tw (M.-H.S.); 2 Department of Radiation Oncology, Taichung Veterans General Hospital, Taichung 40201, Taiwan; bigjohnyou@gamil.com; 3 Institute of Biomedical Science, National Chun-Hsin University, Taichung 402, Taiwan; mlsheu@nchu.edu.tw; 4 Faculty of Medicine, Kaohsiung Medical University, Kaohsiung 80708, Taiwan; a0920732169@gmail.com; 5 Department of Neurosurgery, University of Virginia, Charlottesville, VA 22908, USA; jps2f@hscmail.mcc.virginia.edu; 6 PhD Program in Translation Medicine, Rong Hsing Research Center for Translation Medicine, National Chun-Hsin University, Taichung 402, Taiwan; 7 Department of Medical Research, Taichung Veterans General Hospital, Taichung 40201, Taiwan

**Keywords:** carotid cavernous sinus fistula, gamma knife radiosurgery, adverse effect, cataract, glaucoma, dry eyes

## Abstract

Objective: The leading treatment option for dural carotid–cavernous sinus fistula is an endovascular approach with immediate improvement. Alternatively, radiosurgery is a slow response for obliterating the fistula and poses a radiation risk to the optic apparatus and the associated cranial nerves and blood vessels. In this study, we retrieved cases from a prospective database to assess the ophthalmological outcomes and complications in treating dural carotid cavernous sinus fistula with gamma knife radiosurgery (GKRS). Material and Methods: We retrieved a total of 65 cases of carotid cavernous sinus fistula treated with GKRS with margin dose of 18–20 Gy from 2003 to 2018 and reviewed the ophthalmological records required for our assessment. Results: The mean target volume was 2 ± 1.43 cc. The onset of symptom alleviated after GKRS was 3.71 ± 7.68 months. There were two cases with residual chemosis, two with cataract, two with infarction, one with transient optic neuropathy, and four with residual cranial nerve palsy, but none with glaucoma or dry eyes. In MRA analysis, total obliteration of the fistula was noted in 64 cases with no detectable ICA stenosis nor cavernous sinus thrombosis. In the Cox regression analysis, post-GKRS residual cranial nerve palsy was highly correlated to targeted volume (*p* < 0.05) and age (*p* < 0.05). The occurrence of post-GKRS cataract was related to the initial symptom of chemosis (*p* < 0.05). Conclusion: GKRS for carotid cavernous sinus fistula offers a high obliteration rate and preserves the cavernous sinus vascular structure while conferring a low risk of treatment complications such as adverse radiation risk to the optic apparatus and adjacent cranial nerves.

## 1. Introduction

The dural carotid–cavernous fistula (DCCF) is an acquired vascular abnormality with flow between the cavernous sinus and branches of either external or internal carotid artery [1]. The clinical presentation of DCCF is highly correlated to the venous drainage pattern. For example, symptoms in the orbital or neuroophthalmological presentation are associated with the drainage veins through the superior/inferior ophthalmic, whereas the cortical or deep veins drainage predisposes patients to development of brain stem edema or intracerebral hemorrhage [2,3,4,5].

The leading treatment for DDCF is an endovascular approach, and the transvenous route has high clinical and anatomical cure rates and low incidence of complication [6,7]. In those patients requiring the immediate alleviation of symptoms or harboring the potential risk of brain infarct or hemorrhage, transvenous embolization is typically the first option. However, a transvenous procedure jeopardizes patients with intracerebral hemorrhage through venous rupture or induces cranial nerve palsy due to the embolization materials being sequestered in the cavernous sinus [8,9,10,11,12,13,14,15,16].

Radiosurgery has few serious complications, but the therapeutic effect of radiation needs 3–6 months or longer to afford benefits. Hence, radiosurgery is typically applied to patients with DCCF having mild symptoms or low risk of intracerebral hemorrhage and infarct [17,18,19,20]. The benefits of radiosurgery on DCCF must be weighed against the slow obliteration process and adverse effect of radiation on the optic apparatus and the associated cranial nerve and blood vessels. In this study, we retrieved the data of patients with DCCF treated by GKRS and analyzed their long-term ophthalmological outcomes related to the radiation distributed to lens, lacrimal gland, optic apparatus, intraocular pressure (IOP), cranial nerves, cavernous sinus, and internal carotid artery. Our aim is to gain knowledge of the long-term effects of GKRS on these neurovascular structures and provide a reference on tailoring gamma knife treatment based on the defined benefit-to-risk ratio.

## 2. Materials and Methods

Patient Population

A total of 71 patients with DCCFs were treated by GKRS between September 2003 and August 2018. Their data were retrieved from our gamma knife database. These patients had a detailed examination by ophthalmological test, including visual acuity, measurement of intraocular pressure, and Schirmer’ test, either conducted at referral clinics or at our hospital before GKRS, and then at least one of above-mentioned examinations conducted less than three months before the last OPD follow-up. Excluded from the study were six patients who were either lost in follow-up or had no available ophthalmological records. Finally, we analyzed the remaining 65 cases. The laboratory data on protein C, protein S, prothrombin time, activated partial thromboplastin time, fibrinogen degradation produce, and fibrinogen, as well as cell counts, were collected before GKRS and one year afterward. The study was approved by the Ethical Committee of Taichung Veterans General Hospital (No. CE21156A).

Radiosurgical Technique

After a patient had received a local anesthetic, the Leksell G head frame was affixed to the head, and the patient was monitored for blood pressure, oxygenation, and electrocardiography. For radiosurgical targeting and planning, we used high-resolution biplanar and stereotactic cerebral angiography that included injection where appropriate for selective vertebral artery, internal carotid artery (ICA), and external carotid artery (ECA). Magnetic resonance imaging and MR angiography, including T1-weighted, T2-weighted, time of flight (TOF), spoiled gradient recall, and Gd-enhanced sequence, were obtained to localize the nidus. The MR imaging and cerebral angiography results were transferred to a Leksell GammaPlan station (Elekta Instruments AB). Targets were delineated on the fused MRI and cerebral angiography images. Barrow classification of dural carotid–cavernous fistula (DCCF) include type A, B, C, and D [1]. Type A showed a direct connection between the intra-cavernous internal carotid artery and the cavernous sinus. Type B indicated dural shunt (indirect) between the meningeal branches of the intra-cavernous internal carotid artery and the cavernous sinus. Type C revealed dural shunt (indirect) between meningeal branches of the external carotid artery and the cavernous sinus. Type D consisted of dural shunt (indirect) between both meningeal branches of the intra-cavernous internal carotid artery (type B) and the meningeal branches of the external carotid artery (type C) and the cavernous sinus. Radiosurgery dose plans with single or multiple isocenters were generated to cover the target contour. All patients underwent GKRS with a margin dose of 18–20 Gy prescribed to nidus of 50% to 60% isodose line. All patients were treated with a Leksell gamma knife model D (Elekta AB), primarily by a four-member team consisting of a neurosurgeon, neuroradiologist, radiation oncologist, and medical physicist.

Clinical and Imaging Follow-Up

Patients were followed up in the ophthalmological and neurosurgical outpatient clinics at a 2–3 months interval after GKRS during the first year, and then followed annually thereafter. Clinical data obtained at follow-ups included intraocular pressure, Schirmer’s test, visual acuity, and a detailed neurological examination.

Follow-up MR imaging was performed 3–6 months after GKRS, then at a 6-to-12-month interval until symptoms resolution, and then at a one-to-two-year interval. MR imaging sessions included T1-weighted, T2-weighted, TOF, and Gd-enhancement sequence to compare results with pre-treatment. MR imaging results were used to evaluate the obliteration of the fistula and to detect any unintended postoperative effect as evident on MR images. Additional angiography was performed for the patients whose symptoms did not completely resolve. Images were reviewed by a neuroradiologist, based on the standard criteria [21,22].

Statistical Analyses: Descriptive statistics were recorded according to standard representation, such as mean ± standard deviation or median values and ranges. Factors contributing to cataract, dry eye, increased intraocular pressure, imaging alteration, and neurological outcome were assessed with the Mann–Whitney test, chi-square test, and Fisher’s exact test. Cox regression test was used to determine risk factors related to cataract, brain infarction, dry eye, cranial nerve palsy, and imaging alteration. Statistical significance was set at *p*-value < 0.05.

## 3. Results

### 3.1. General Data

The patients had a mean age of 60.8 ± 14.9 years old, and a female-to-male ratio of 43/22. Their mean duration from onset of symptoms to treatment was 4.77 ± 6.31 months. History of trans-arterial embolization was found in seven cases. Their mean follow-up period post-GKRS was 97.7 ± 52.9 months.

Laboratory data regarding protein C, protein S, prothrombin time, activated partial thromboplastin time, fibrinogen degradation produce, and fibrinogen were all within normal limits before GKRS (data not shown). Their mean WBC was 7160 ± 1294/μL with a neutrophil/lymphocyte ratio of 2.68 ± 0.56 before GKRS.

Clinical presentations included 60 patients (92.3%) patients with exophthalmos either combined with either cranial nerve palsy (16 patients, 24.6%) or increased intra-ocular pressure (26 patients, 40%), and only 5 patients had solitary cranial nerve palsy. Ophthalmological presentations included 17 cases on the right side, 37 cases on the left side, and 11 cases on both sides. The locations of fistula as shown in cerebral angiography were as follows: 23 on the right sides, 26 on the left sides, 16 on both sides. We categorized our cases into the Barrow and Cognard’s classification to investigate the outcome related to the classification [1,2]. The barrow classification included 59 in type D, 2 in type C, and 4 in type B. According to Cognaed’s classification, there were 12 cases of grade I, 3 cases of grade II, 42 cases of grade IIa, and 8 cases of grade IIb. Among the eight cases of grade IIb, seven cases showed the illustration of cortical veins in a very late arterial phase, and only one case showed early filling of the cortical vein, which revealed no obliteration of the fistula.

The GKRS treatment parameters included target volume 2 ± 1.43 cc, margin dose of 18 ± 0.79 Gy, isodose line of 55 ± 5.5%, conformity index of 1.28 ± 0.08, and number of isocenter of 5.4 ± 3.6. All details are summarized in Table 1.

### 3.2. Treatment Parameters

IOP before GKRS was 17 ± 7.61 mmHg, and Schirmer’s test was 6.97 ± 0.88 mm. The treatment parameters included the dosage of lens (right 0.43 ± 0.33 Gy, left 0.44 ± 0.28 Gy), lacrimal gland (right 0.45 ± 0.31 Gy, left 0.46 ± 0.21 Gy), optic nerve (right 3.84 ± 1.75 Gy, left 3.90 ± 1.8 Gy), chiasma (2.6 ± 0.96 Gy), pituitary stalk (1.94 ± 0.84 Gy), lateral wall of cavernous sinus (right 9.51 ± 5.51 Gy, left 11.74 ± 5.62 Gy), internal carotid artery (14.92 ± 14.58% of right ICA > 20 Gy, 20.59 ± 13.14 % of left ICA > 20 Gy), and brain stem (6.6 ± 2.7 Gy), which are shown in Table 2.

### 3.3. Treatment Outcome

The duration of symptom alleviation was 3.71 ± 7.68 months post GKRS. The treatment outcomes and associated complications are depicted in Table 3.

**Figure 1:**
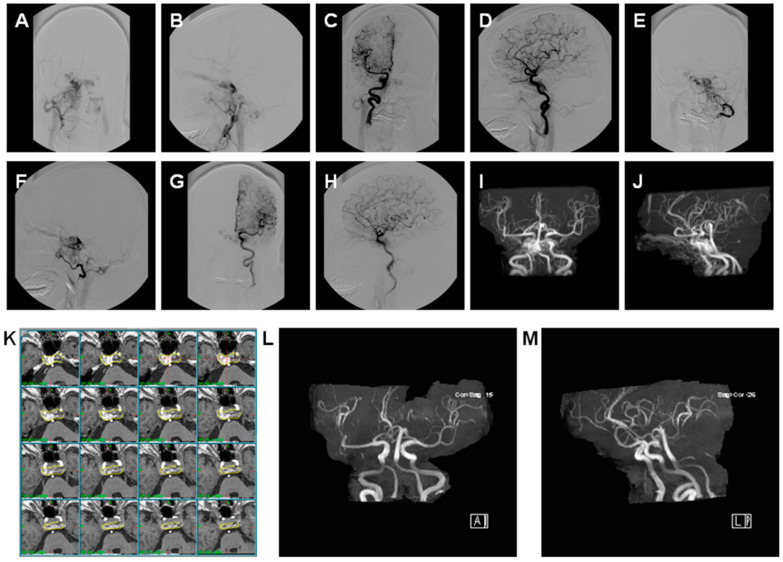
Illustration of bilateral carotid cavernous sinus fistula targeted on bilateral cavernous sinus by GKRS with total obliteration of the fistula but without the recovery of cranial nerve function. A 73-year-old female suffered acute onset of bilateral chemosis and bilateral 6th nerve palsy treated with gamma knife radiosurgery with residual bilateral 6th nerve palsy 10 years after GKRS. (**A**) Cerebral angiography in PA view in right ECA injection. (**B**) Cerebral angiography in lateral view in right ECA injection. (**C**) Cerebral angiography in PA view in right ICA injection. (**D**) Cerebral angiography in lateral view in right ICA injection. (**E**) Cerebral angiography in PA view in left ECA injection. (**F**) Cerebral angiography in lateral view in left ECA injection. (**G**) Cerebral angiography in PA view in left ICA injection. (**H**) Cerebral angiography in lateral view in left ICA injection. (**I**) Cerebral MAR in PA view. (**J**) Cerebral MRA in lateral view. (**K**) Demonstration of GKRS with treated volume of 3.6 cc in 18 Gy (50% line), yellow line: 50% line. (**L**) Cerebral MRA in PA view 10 years after GKRS. (**M**) Cerebral MRA in lateral view 10 years after GKRS. ECA, ICA, and MRA: see text.

**Figure 2:**
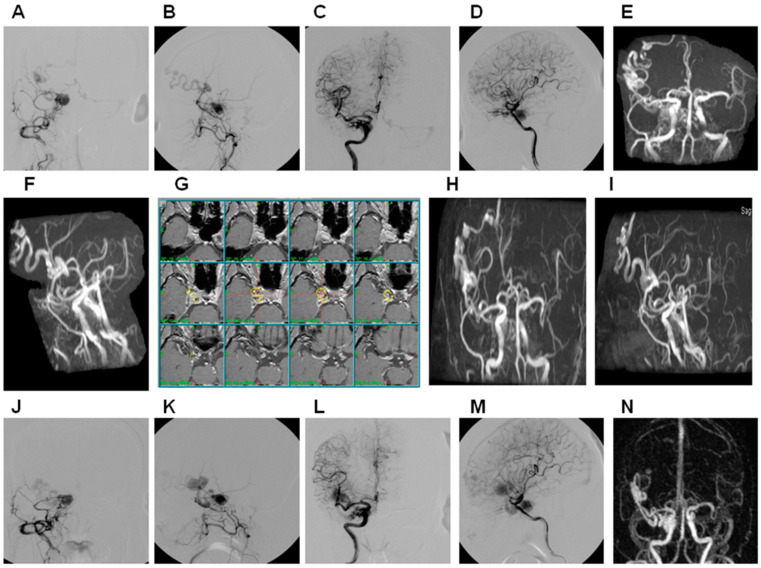
Illustration of failed treatment of right carotid cavernous sinus by GKRS. A 68-year-old female presented with right eye chemosis and increased intraocular pressure for 2 months and received gamma knife treatment targeted at the cavernous sinus on the right side without obliteration of the fistula. (**A**) Cerebral angiography in PA view in right ECA injection. (**B**) Cerebral angiography in lateral view in right ECA injection. (**C**) Cerebral angiography in PA view in right ICA injection. (**D**) Cerebral angiography in lateral view in right ICA injection. (**E**) Cerebral MAR PA view. (**F**) Cerebral MRA in lateral view. (**G**) Demonstration of GKRS with treated volume of 0.518 cc in 18 Gy (55% line), yellow line: 55% line. (**H**) Cerebral MRA in PA view 6 years after GKRS. (**I**) Cerebral MRA in lateral view 6 years after GKRS. (**J**) Cerebral angiography in PA view in right ECA injection 6 years after GKRS. (**K**) Cerebral angiography in lateral view in right ECA injection 6 years after GKRS. (**L**) Cerebral angiography in PA view in right ICA injection 6 years after GKRS. (**M**) Cerebral angiography in lateral view in right ICA injection 6 years after GKRS. (**N**) Cerebral MRA in PA view 8 years after GKRS. ECA, ICA, and MRA: see text.

**Figure 3:**
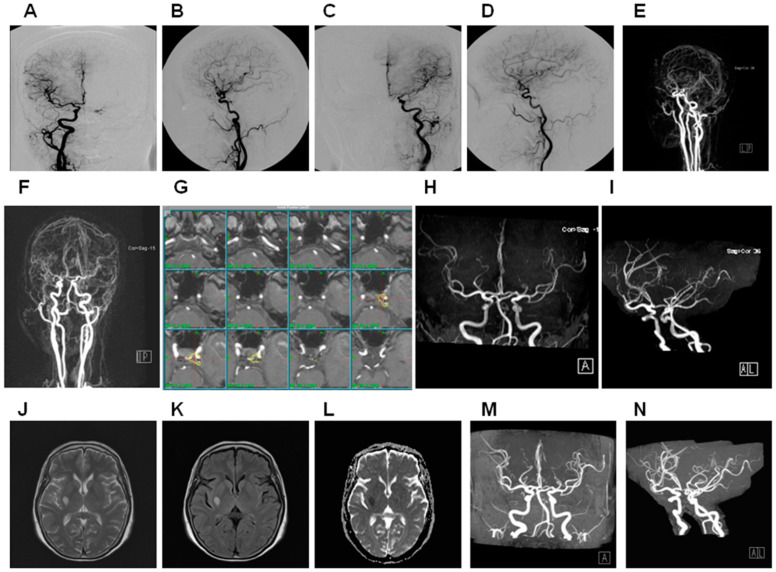
Illustration of right carotid cavernous sinus fistula treated by GKRS with late onset of brain infarct. A 52-year-old female suffered left abducense nerve palsy for 5 months and received GKRS targeted on left cavernous sinus. The total obliteration of the fistula and recovery of nerve function was noted. However, she suffered right-side putamen infarct, which was not related to irradiation to the right internal carotid artery. (**A**) Cerebral angiography in PA view in right CCA injection. (**B**) Cerebral angiography in lateral view in right CCA injection. (**C**) Cerebral angiography in PA view in left CCA injection. (**D**) Cerebral angiography in lateral view in left CCA injection. (**E**) Cerebral MAR lateral view. (**F**) Cerebral MRA in PA view. (**G**) Demonstration of GKRS with treated volume of 0.21 cc in 20 Gy (50% line), yellow line: 50% line. (**H**) Cerebral MRA in PA view 2 years after GKRS. (**I**) Cerebral MRA in lateral view 2 years after GKRS. (**J**) MRA in T2 weight showed a hypersignal lesion over right putamen 13 years after GKRS. (**K**) MRA in FLAIR showed a hypersignal lesion over right putamen 13 years after GKRS. (**L**) MRA in DWI showed a hypo-signal lesion over right putamen 13 years after GKRS. (**M**) MRA in PA view showed no definite carotid stenosis 13 years after GKRS. (**N**) MRA in lateral view showed no definite carotid stenosis 13 years after GKRS. ECA, ICA, and MRA: see text. CCA: common carotid artery; FLAIR: fluid-attenuated inversion recovery; DWI: diffusion-weighted magnetic resonance imaging.

We found two cases (3.1%) with residual red eyes, and four cases (6.2%) with cranial nerve palsy, but no cases of dry eyes or glaucoma. In MRA assessment, 64 of 65 cases (98.4%) showed total obliteration of the fistula (Figure 1), and 1 case showed residual fistula but was given no further treatment due to old age (Figure 2). All 65 cases of MRA studies showed preservation of bilateral cavernous sinus, and no case demonstrated evidence of ICA stenosis. IOP at the last OPD was 13.08 ± 1.4 mmHg, and Schirmer’s test was 6.89 ± 0.99 mm. The long-term complications were as follows: two cases with brain infarction (Figure 3), two cases with cataract, four cases with cranial nerve palsy (Figure 1), and one case with transient optic neuropathy.

Lab data showed after GKRS a reduction of WBC of 5902.31 ± 1357.26/μL (*p* < 0.001) and an elevated ratio of neutrophil to lymphocytes at 5.07 ± 27.71 (*p* < 0.001).

### 3.4. Risk Factor Analysis Contributing to Adverse Effect

In the Cox regression model, cataract formation was highly correlated with the presenting symptom of red eyes (*p* < 0.05) (Table 4). The post-GKRS residual cranial nerve palsy was highly correlated with GKRS treatment volume (*p* < 0.05) and with age (*p* < 0.05) at GKRS (Table 5). The logistic regression included in Supplemental Table S1 shows that the residual cranial nerve palsy was highly correlated to the target on bilateral cavernous sinus (*p* < 0.05) and the number of isocenter (*p* < 0.05). We found no significant risk factors for development of post-GKRS brain infarction.

## 4. Discussion

While embolization is the gold standard treatment of most indirect C-C fistula, radiosurgery is a reasonable alternative for patients without aggressive C-C fistula behavior. Such radiosurgery typically has a slow response with rare adverse radiation effects on the optic apparatus and adjacent cranial nerve and blood vessel structures. In some cases, radiosurgery exerts a high obliteration rate and preserves the patency of cavernous sinus with minimal injury to the associated cranial nerve and blood vessels. In long-term follow-up, few patients develop cataract formation, while none develop glaucoma or dry eyes. It seems that radiosurgery is a useful tool in the treatment armamentarium for managing indirect C-C fistula and exhibits few adverse side effects.

Symptoms from chemosis and proptosis are usually relieved after obliterating DCCF. However, a significant proportion of post-GKRS patients with cranial nerve palsy do not recover their normal neurological function, a finding compatible with the transvenous embolization [8,9,10,11,12,13,14,15,16]. Importantly, the new onset of cranial nerve palsy in a transvenous embolization is not observed during radiosurgery [16]. Except for the slow resolution of symptoms and signs, radiosurgery appears to be an attractive option for treating DCCF, especially for those patients without immediate life-threatening conditions or neurological risks.

Cerebral angiography offers dynamic aspects of the cerebral vasculature from multiple projection angles independent of blood flow velocity. It is the gold standard to evaluate the obliteration of DCCFs. Given the risk of complications from the procedure of cerebral angiography and a low risk of residual fistula, we used primarily noninvasive MR imaging and/or MR angiography during follow-ups. The obliteration rate of DCCF assessed by MR angiography may, however, be overestimated [2]. Angiography has a higher detection rate for DCCF when compared with MR imaging [17,23]. In contrast, the TOF MR sequence is equally as sensitive as conventional angiography, but it might have a higher false-positive rate [22,24]. In many cases, often due to the reluctance of the patients or their family to undergo invasive testing, we did not perform angiography for final obliteration confirmation. However, our MR angiography/MR imaging probably provided reliable information for assessing the obliteration of DCCF and treatment-related complications. Moreover, it seems unlikely that any such undetected residual AVF on MRI/A meaningfully contributed to lingering neurological sign or symptoms in such patients.

Radiosurgery produces radiation effects on the blood vessel wall, and consequently leads to the progressive intimal hypertrophy and thrombosis of the DCCF [4,5,17,19]. The alleviation of symptoms in our patients typically started 2–3 months after radiosurgery, and MR imaging revealed the fistula regression in 6 to 9 months. Such a finding is compatible with the published literature [5,17,18]. Symptoms of chemosis and proptosis of our patients were typically relieved after obliteration of the DCCF. However, a significant proportion of the cranial nerve palsy patients did not return to normal functioning. This finding is compatible with the reports on transvenous embolization [6,7,16] or radiosurgical treatment [17,19,20]. However, the new onset of cranial nerve palsy often found following a transvenous embolization was not observed following treatment with radiosurgery in our patients [16]. Except for the slowness of the alleviation of symptoms and signs, radiosurgery seems to be an attractive alternative treatment for DCCF, especially for patients without an immediate life-threatening condition.

In radiosurgery, the steep dose gradient and high accuracy of imaging protect the optic apparatus and brainstem from high-dose irradiation [17]. In the current study, the optic apparatus received a radiation dose <8 Gy, which is generally regarded as safe for the optic pathways. Except for one case with transient optic neuritis, which has been published before [5], we found no long-lasting optic neuropathy in our study. Other cranial nerves in the cavernous sinus are in general more resistant to injury from radiation when compared with the optic nerve. These nerves were reported to tolerate up to 40 Gy in a single fraction [25]. In the present study, the maximum dose delivered to the cavernous sinus wall was 20 Gy, a dose markedly <40 Gy. We also found no new onset of cranial nerve palsy during our long-term follow-up. In the univariate analysis, higher incidence of residual cranial palsy was significantly correlated with old age and radiation volume (all four cases harboring residual cranial nerve palsy targeted on bilateral cavernous sinus due to involvement of both sides). It is likely that refining the dose planning could greatly reduce the radiation exposure to cranial nerves. How to optimally plan such cases properly remains an important goal for the avoidance of radiosurgical complication.

The increase of IOP in CCF is mainly due to the increased episcleral and vortex vein pressures. In such cases, closure of the fistula and normalization of circulation result in lowering the IOP. In other cases, glaucoma might be caused by iris neovascularization due to reduced retinal perfusion or vascular engorgement and edema of the choroid and ciliary body. The result is a forward movement of the iris/lens leading to a pupil block glaucoma [26,27]. In the occurrence of glaucoma caused by the SRS, the dose to the above-mentioned structure may exceed 13 to 20 Gy [28,29]. In our study, the dose to these vital structures was much less <13 Gy. Such a low dose likely explains why none of our patients had developed glaucoma after gamma knife treatment.

Anecdotal studies reported that radiosurgery causes carotid stenosis in patients harboring a cavernous sinus meningioma or pituitary adenoma [30,31,32]. However, very little information is available on the actual radiation dose the affected arteries have been exposed to. In such a case, the radiation dose exposed to the carotid artery ranges from 25 to 30 Gy and even as high 40 Gy, a level that generates risk for the developing carotid artery stenosis [33,34,35]. According to other studies, a heterogeneous dose with a hot spot targeted on the carotid artery may pose a risk for carotid artery stenosis [35,36]. In the current study, the radiation dose to the carotid artery was much <30 Gy which is considered a detrimental factor for carotid stenosis [34]. Furthermore, the DDCF presented the intact stroma of a vessel refractory to radiation injury that was in contrast with patients harboring the carotid artery invaded by a tumor [37]. In the current study, there were two cases of brain infarction without definite carotid stenosis after GKRS. These cases are different from the reports on stroke related to carotid stenosis. Due to the rarity of these phenomena, we recommend periodical examination of MRI/MRA, which is essential for patients with DDCF after GKRS.

Radiation-induced lacrimal gland atrophy with consequent reduction in tear volume can be caused by a single dose of 20 Gy or higher [38]. At doses of 30–45 Gy, severe dry eyes occur at 19% incidence, while at doses <30 Gy, no dry eye occurs [39]. In GKRS, the median dosage of 4 Gy or higher delivered to the lacrimal gland causes a high incidence of dry eyes [40]. It is well known that radiation-induced dry eyes is a multifactorial condition, and the contributing factors are likely patient age, female gender, and systemic co-morbidities such as hypertension, diabetes, and medication use [41,42,43]. In this cohort, the dosage to the lacrimal gland was far less than that those in the literature. Such a low dose may account for the absences of dry eyes reported in our patients.

The onset of radiation-induced cataract is extremely common, occurring at latency of 2 or 3 years, with an interval of 6 to 64 months [44]. Taking into account the wide range of dose and fractionation (from 8% to 83% depending on the characteristics of the treatment), it is difficult to estimate the actual incidence of radiation-induced cataract [45]. In general, a maximum of 2 Gy on the lens is the critical dosage for developing cataract [45]. However, in some studies, radiation dosage as low as 0.5 Gy still has a detrimental effect on cataracts development [46,47]. Therefore, radiation exposure during gamma knife treatment or cerebral angiography carries a higher risk for cataractogenesis matched to the control [48]. In this study, the lens dose was far less than 0.5 Gy, which is believed to be the threshold for cataract development. However, two of our patients still developed symptomatic cataracts. Such cataract formation was highly correlated to their original symptom of chemosis. This result failed to be explained solely by radiation exposure.

Our present results showed an excellent obliteration rate with little radiation adverse effects after properly adjusted delivery of radiation to vulnerable structures. However, there were several limitations in this study. First, the case number is small, which reduces the statistical power of the analysis. Second, data in ophthalmic examination were obtained from different sources (including medical centers or private clinics) without the standard criteria to assess the potential adverse effects. Even with some flaws in this study, DDCF treated with GKRS likely affords a high obliteration rate without increasing the additional adverse response. It is suggested that radiosurgery for DCCFs could be considered in patients without seriously increased intraocular pressure and neuroimaging without aggressive venous cortical reflux. In addition, radiosurgery for residual DDCF after failed endovascular attempt was also a reasonable approach.

## 5. Conclusions

For patients harboring DDCF without immediate life-threatening symptoms, GKRS is likely an effective treatment and carries a long-term low adverse effect to the nearby optic apparatus and vascular structures. With careful case selection and proper dose planning, GKRS is an effective treatment option in DDCF.

## Figures and Tables

**Table 1:** Demography of the patients (*n* = 65).

		Mean ± SD
Age		60.8 ± 14.9
Sex	M	22 (33.8%)
	F	43 (66.2%)
WBC/ul		7160 ± 1294
N/L (ratio)		2.68 ± 0.56
History of embolization		7 (10.8%)
Follow-up periods (months)		97.7 ± 52.9
Onset to treatment (months)		4.77 ± 6.31
Symptom/signs	Red eye	60 (92.3%)
	Increases IOP	26 (40%)
	Cranial nerve palsy	21 (32.7%)
Location of symptom	Right	17 (26.2%)
	Left	37 (56.9%)
	Bilateral	11 (16.9%)
Location of fistula	Right	23 (35.4%)
	left	26 (40%)
	bilateral	16 (24.6%)
Barrow classification	B	4 (6.1%)
	C	2 (3.1%)
	D	59 (90.8%)
Target volume (cc)		2 ± 1.43
Margin dose (Gy)		18 ± 0.79
Maximum dose (Gy)		32.75 ± 3.65
Maximum error in angiography (mm)		0.12 ± 0.04
Isodose line (%)		55 ± 5.5
Conformity index		1.28 ± 0.08
Number of isocenters		5.4 ± 3.6

Data are presented as mean± standard deviation. N/L: neutrophil/lymphocyte.

**Table 2:** Ophthalmological test and radiation dosage in the optic apparatus and adjacent structure.

	Mean ± SD
Right optic nerve (Gy)	3.84 ± 1.75
Left optic nerve (Gy)	3.90 ± 1.8
Chiasma (Gy)	2.6 ± 0.96
Right lens (Gy)	0.43 ± 0.33
Left lens (Gy)	0.44 ± 0.28
Right lacrimal gland (Gy)	0.45 ± 0.31
Left lacrimal gland (Gy)	0.46 ± 0.21
Brain stem (Gy)	6.6 ± 2.7
Pituitary stalk (Gy)	1.94 ± 0.84
Right lateral cavernous sinus wall (Gy)	9.51 ± 5.51
Left lateral cavernous sinus wall (Gy)	11.74 ± 5.62
Right ICA volume (cc)	0.46 ± 0.03
Left ICA volume (cc)	0.45 ± 0.02
% of right ICA > 20 Gy	14.92 ± 14.58
% of left ICA > 20 Gy	20.59 ± 13.14
IOP (mmHg)	17 ± 7.61
Schirmer’s test (mm)	6.97 ± 0.88

Data are presented as mean± standard deviation. ICA: internal carotid artery; IOP: intraocular pressure.

**Table 3:** Outcome of C-C fistula (*n* = 65).

		Mean ± SD
Duration of S/S alleviated (months)		3.71 ± 7.68
MRA outcome	Obliteration	64 (98.4%)
	Preservation of cavernous sinus	65 (100%)
	ICA stenosis	0
Residual symptom	Red eye	2 (3.1%)
	Cranial nerve palsy	4 (6.2%)
	Glaucoma	0
	Dry eyes	0
Last opththmalogical test	Schirmer’s test (mm)	6.89 ± 0.99
	IOP (mmHg)	13.08 ± 1.4
Complication post GKRS	Infarction	2 (3.1%)
	Cataract	2 (3.1%)
	Transient optic nerve neuropathy	1 (1.5%)

Data are presented as mean± standard deviation.

**Table 4:** Risk factors analysis for cataract development after GKRS.

	Univariate		
	HR	95%CI	*p* Value
N/L (Pre-GKRS)	2.18	(0.14–35.22)	0.583
S/S (Yes vs. No)			
Red eye	0.06	(0.00–0.97)	0.047 *
Increase IOP	1.38	(0.08–22.84)	0.823
Cranial nerve palsy	1.57	(0.10–25.73)	0.752
Tumor volume (cc)	0.76	(0.19–3.04)	0.701
Number of isocenters	0.97	(0.66–1.42)	0.880
Maximum dose (Gy)	0.79	(0.56–1.12)	0.188
Peripheral isodose line (%)	1.15	(0.94–1.40)	0.170
Margin dose (Gy)	0.93	(0.19–4.52)	0.927
Rt optic nerve dosage (Gy)	1.55	(0.69–3.51)	0.289
Lt optic nerve dosage (Gy)	1.55	(0.68–3.50)	0.296
Chiasma dosage (Gy)	1.25	(0.34–4.60)	0.742
Pituitary stalk dosage (Gy)	2.40	(0.52–11.09)	0.261
Brain stem dosage (Gy)	0.46	(0.18–1.18)	0.106
Rt lens dosage (Gy)	0.00	(0.00–746.84)	0.366
Lt lens dosage (Gy)	0.40	(0.00–103.35)	0.747
Rt Lacrimal gland dosage (Gy)	0.00	(0.00–746.84)	0.366
Lt Lacrimal gland dosage (Gy)	0.40	(0.00–103.35)	0.747
Rt lateral cavernous sinus wall dosage (Gy)	1.14	(0.83–1.57)	0.414
Lt lateral cavernous sinus wall dosage (Gy)	1.00	(0.76–1.32)	0.987
IOP at GKRS (mmHg)	1.04	(0.89–1.22)	0.612
Schirmer’s test at GK (mm)	1.36	(0.23–7.89)	0.733
Percentage of Rt ICA > 20 Gy (%)	1.03	(0.94–1.13)	0.541
Percentage of Rt ICA (>20 Gy vs. ≤20 Gy)	1.74	(0.11–28.27)	0.696
Percentage of Lt ICA > 20 Gy (%)	0.96	(0.85–1.08)	0.526
Percentage of Lt ICA (>20 Gy vs. ≤20 Gy)	0.74	(0.05–11.89)	0.832

Cox regression. * *p* < 0.05, Rt = right; Lt = left.

**Table 5:** Risk factors analysis for cranial nerve palsy after GKRS.

	Univariate		
	HR	95%CI	*p* Value
age	1.20	(1.03–1.40)	0.022 *
Sex			
Male	ref.		
Female	0.56	(0.05–6.42)	0.643
WBC (Pre GKRS)/μL	1.00	(1.00–1.00)	0.061
Tumor volume (cc)	2.09	(1.04–4.19)	0.038 *
Number of isocenter	1.17	(0.90–1.51)	0.235
Maximum dose (Gy)	1.07	(0.81–1.42)	0.632
Peripheral isodose line (%)	0.94	(0.77–1.16)	0.561
Margin dose (Gy)	0.79	(0.23–2.73)	0.704
Rt optic nerve dosage (Gy)	1.53	(0.84–2.79)	0.167
Lt optic nerve dosage (Gy)	1.18	(0.65–2.17)	0.585
Chiasma dosage (Gy)	1.40	(0.56–3.48)	0.470
Pituitary stalk dosage (Gy)	1.90	(0.62–5.84)	0.262
Brain stem dosage (Gy)	1.18	(0.82–1.71)	0.378
Rt lateral cavernous sinus wall dosage (Gy)	1.43	(0.99–2.07)	0.060
Lt lateral cavernous sinus wall dosage (Gy)	1.02	(0.82–1.27)	0.888
IOP at GK (mmHg)	1.30	(0.87–1.94)	0.205
Schirmer’s test at GKRS (mm)	0.53	(0.15–1.88)	0.328
Percentage of Rt ICA > 20 Gy (%)	1.05	(0.98–1.12)	0.140
Percentage of Lt ICA > 20 Gy (%)	1.04	(0.96–1.13)	0.339
Percentage of Lt ICA (>20 Gy vs. ≤20 Gy)	0.70	(0.10–5.16)	0.730

Cox regression. * *p* < 0.05, Rt = right; Lt = left.

## Data Availability

Not applicable.

## Supplementary Materials

The following supporting information can be downloaded at: https://www.mdpi.com/article/10.3390/life12081175/s1, Table S1: Risk factors analysis for cranial nerve palsy after GKRS.
