# Supplementary material for: Gamma Knife Radiosurgery for Indirect Dural Carotid–Cavernous Fistula: Long-Term Ophthalmological Outcome
Source: Life (Basel). 2022 Aug 1;12(8):1175. doi: 10.3390/life12081175 (PMC9410130; doi:10.3390/life12081175)
Supplement: Supplementary file 1 [file life-12-01175-s001.zip › life-1756252-supplementary.pdf]

Supplemental Table S1: Risk factors analysis for cranial nerve palsy after GKRS

| | Univariate | | Multivariate | |
|---|---|---|---|---|
| | OR (95%CI) | *p* value | OR (95%CI) | *p* value |
| age | 1.08 (0.98-1.19) | 0.125 | | |
| Sex | | | | |
| Male | ref. | | | |
| Female | 1.57 (0.15-16.09) | 0.702 | | |
| WBC (Pre GKRS) (/μl) | 1.001 (0.9999-1.001) | 0.067 | | |
| N/L (Pre GKRS) (%) | 8.3 (0.84-82.06) | 0.070 | | |
| Number of target | | | | |
| Single cavernous sinus | ref. | | | |
| Bilateral cavernous sinus | 12.25 (1.17-128.39) | 0.037* | 5.35 (0.39-73.72) | 0.210 |
| Tumor volume (cc) | 1.44 (0.81-2.56) | 0.210 | | |
| Number of isocenter | 1.38 (1.03-1.83) | 0.028* | 1.25 (0.91-1.72) | 0.171 |
| Maximum dose (Gy) | 1.17 (0.86-1.60) | 0.312 | | |
| Peripheral isodose (%) | 0.90 (0.71-1.14) | 0.373 | | |
| Margin dose (Gy) | 0.95 (0.26-3.48) | 0.935 | | |
| Rt optic nerve dosage (Gy) | 1.34 (0.77-2.34) | 0.295 | | |
| Lt optic nerve dosage (Gy) | 0.93 (0.52-1.68) | 0.818 | | |
| Chiasma dosage (Gy) | 1.39 (0.51-3.80) | 0.523 | | |
| Pituitary stalk dosage (Gy) | 1.59 (0.53-4.79) | 0.409 | | |
| Brain stem dosage (Gy) | 1.42 (0.92-2.19) | 0.117 | | |
| Rt lens dosage (Gy) | 6.39 (0.73-55.55) | 0.093 | | |
| Lt lens dosage (Gy) | 7.64 (0.47-123.74) | 0.153 | | |
| Rt Lacrimal gland dosage (Gy) | 6.39 (0.73-55.55) | 0.093 | | |
| Lt Lacrimal gland dosage (Gy) | 7.64 (0.47-123.74) | 0.153 | | |
| Rt lateral Cavernous sinus wall dosage (Gy) | 1.14 (0.94-1.38) | 0.183 | | |
| Lt lateral Cavernous sinus wall dosage (Gy) | 0.93 (0.77-1.12) | 0.418 | | |
| IOP at GKRS (mmHg) | 1.53 (0.76-3.07) | 0.236 | | |
| Schirmer's test at GKRS (mm) | 1.49 (0.46-4.86) | 0.510 | | |
| Percentage of Rt ICA 20Gy (%) | 1.07 (0.99-1.15) | 0.105 | | |
| Percentage of Rt ICA (>20Gy vs <=20Gy) | 1.54 (0.20-11.69) | 0.675 | | |
| Percentage of Lt ICA 20Gy (%) | 1.03 (0.95-1.12) | 0.518 | | |
| Percentage of Lt ICA (>20Gy vs <=20Gy) | 0.53 (0.07-4.00) | 0.534 | | |
| Embolization (Yes vs No) | 3.06 (0.27-34.19) | 0.365 | | |

Logistic regression. *$p$<0.05, **$p$<0.01.
